# Evacuation after the Fukushima Daiichi Nuclear Power Plant Accident Is a Cause of Diabetes: Results from the Fukushima Health Management Survey

**DOI:** 10.1155/2015/627390

**Published:** 2015-05-27

**Authors:** Hiroaki Satoh, Tetsuya Ohira, Mitsuaki Hosoya, Akira Sakai, Tsuyoshi Watanabe, Akira Ohtsuru, Yukihiko Kawasaki, Hitoshi Suzuki, Atsushi Takahashi, Gen Kobashi, Kotaro Ozasa, Seiji Yasumura, Shunichi Yamashita, Kenji Kamiya, Masafumi Abe

**Affiliations:** ^1^Department of Nephrology, Hypertension, Diabetology, Endocrinology, and Metabolism, Fukushima Medical University, Fukushima 960-1295, Japan; ^2^Radiation Medical Science Center for the Fukushima Health Management Survey, Fukushima Medical University, Fukushima 960-1295, Japan; ^3^Department of Epidemiology, Fukushima Medical University, Fukushima 960-1295, Japan; ^4^Department of Pediatrics, Fukushima Medical University, Fukushima 960-1295, Japan; ^5^Department of Radiation Life Sciences, Fukushima Medical University, Fukushima 960-1295, Japan; ^6^Department of Radiation Health Management, Fukushima Medical University, Fukushima 960-1295, Japan; ^7^Department of Cardiology and Hematology, Fukushima Medical University, Fukushima 960-1295, Japan; ^8^Department of Gastroenterology and Rheumatology, Fukushima Medical University, Fukushima 960-1295, Japan; ^9^Department of Planning and Management, National Institute of Radiological Sciences, Fukushima Medical University, Fukushima 960-1295, Japan; ^10^Department of Epidemiology, Radiation Effects Research Foundation, Fukushima Medical University, Fukushima 960-1295, Japan; ^11^Department of Public Health, Fukushima Medical University, Fukushima 960-1295, Japan; ^12^Atomic Bomb Disease Institute, Nagasaki University, Nagasaki, Japan; ^13^Research Institute for Radiation Biology and Medicine, Hiroshima University, Hiroshima, Japan

## Abstract

The Great East Japan Earthquake and Fukushima Daiichi nuclear disaster in 2011 forced the evacuation of a large number of residents and created changes in the lifestyle of the evacuees. These changes may have affected the evacuees' glucose metabolism, thereby leading to an increase in the incidence of diabetes. This study included Japanese men and women who were living near the Fukushima Daiichi Nuclear Power Plant in Fukushima prefecture before the disaster. Subjects subsequently underwent annual health checkups with a focus on metabolic syndromes, which were conducted under the Health Care Insurers. Using the Comprehensive Health Check survey, we analyzed changes in the glucose metabolism before and after the disaster. A total of 27,486 subjects underwent follow-up examinations after the disaster, with a mean follow-up period of 1.6 years. After the disaster, the prevalence of diabetes increased significantly, and we observed that the incidence of diabetes was significantly greater among evacuees than among nonevacuees. Furthermore, multivariate logistic regression analysis revealed that evacuation was significantly associated with the incidence of diabetes. In conclusion, this is the first study to demonstrate that evacuation is associated with the incidence of diabetes. This information may be used to guide follow-up recommendations for evacuees.

## 1. Introduction

On March 11, 2011, an earthquake (the Great East Japan Earthquake) occurred off the eastern cost of Japan. It generated a tsunami, which struck the Tokyo Electric Power Company's Fukushima Daiichi Nuclear Power Plant, and subsequently released radiation into the Fukushima prefecture. The resulting radiation levels forced the evacuation of a number of nearby towns, which caused significant changes to the lifestyle of the evacuees and anxiety about the radiation effects. Shortly after the disaster, the Fukushima prefecture launched the Fukushima Health Management Survey to investigate the effects of the long-term, low-dose radiation exposure caused by the disaster [1]. The survey comprises a basic survey, which estimates the individual radiation exposure for each resident and four detailed surveys, which include a Comprehensive Health Check, thyroid ultrasonography, mental health and lifestyle survey, and a survey of pregnant women and nursing mothers [1]. The Comprehensive Heath Check was implemented to facilitate the early detection and treatment of radiation-related disease as well as help prevent lifestyle-related diseases, such as diabetes, hypertension, dyslipidemia, obesity, and cardiovascular diseases.

Insulin resistance is defined as a decreased ability of target tissues such as fat, liver, and skeletal muscle to respond to insulin and is a characteristic feature of type 2 diabetes. Unfortunately, the development of insulin resistance is often a result of lifestyle factors such as poor diet, lack of exercise, smoking, and stress, many of which are encountered in the postdisaster setting. Although genetic factors contribute to the development of insulin resistance, it is partially preventable, as a number of the lifestyle factors can be modified to decrease their contribution. Medical interventions can also decrease insulin resistance and preserve or improve *β*-cell function and are typically effective in slowing the progression from impaired glucose intolerance to diabetes or even in reversing intolerance to normal glucose tolerance. In addition, many interventions that improve insulin resistance can also affect the secretion of insulin. Therefore, although disasters can increase the incidence of insulin resistance, the incidence of type 2 diabetes can be reduced by providing evacuees with appropriate support and medication.

The primary goals of the Fukushima Health Management Survey were to monitor the long-term health of the affected residents, promoting their future well-being, and to determine whether long-term, low-dose radiation exposure had significant adverse health effects. After the disaster, residents of the government-designated evacuation zone were forced to change their lifestyle, diet, exercise, and other personal habits. In addition, many did not receive adequate health checkups and experienced stress regarding their health. The Comprehensive Heath Check survey attempted to review the residents' health information, while also assessing the incidence of various diseases among the evacuees. In this study, we used the information obtained from the Fukushima Health Management Survey to identify changes in subjects' glucose metabolism before and after the disaster.

## 2. Materials and Methods

### 2.1. Subjects and Study Design

The Fukushima Health Management Survey was carried out by researchers from Fukushima Medical University. Subjects included in the survey were Japanese men and women who were living near the Fukushima Daiichi Nuclear Power Plant (radius, 20 km) in Fukushima prefecture at the time of the disaster and who were residents of Tamura, Minami-Soma, Kawamata, Hirono, Naraha, Tomioka, Kawauchi, Okuma, Futaba, Namie, Katsurao, Iitate, and Date. In 2010, the census populations of these communities were 42,085, 71,661, 16,065, 5,495, 7,927, 15,854, 3,074, 11,553, 7,171, 21,551, 1,582, 6,584, and 67,684 (total, 278,276), respectively. All residents of Hirono, Naraha, Tomioka, Kawauchi, Okuma, Futaba, Namie, Katsurao, and Iitate, as well as many residents of Tamura, Minami-Soma, Kawamata, and Date, were forced to evacuate their homes after the disaster. Since 2008, residents from these communities who are aged ≥40 years have undergone annual health checkups by the National Health Care Insurers with a focus on detecting metabolic syndromes. The target population for checkups consisted of 91,554 men and women in 2010. Between 2008 and 2010, 41,633 subjects (18,745 men and 22,888 women; mean age, 67 years) from these communities participated in the health checkups.

Annual health checkups were also conducted in 2011 and 2012 as part of the Comprehensive Health Check survey. The detailed methods for this survey have been reported previously [1]. In brief, the health checkup consisted of examinations of all officially registered residents who were living in the evacuation zone at the time of the earthquake. However, only residents aged 40–90 years were included in our analyses. The initial exclusion criterion for our study was missing hemoglobin A1c (HbA1c) data or missing information regarding the use of antihyperglycemic drugs. The remaining data were then used as baseline data for our analyses. After the exclusions, 27,486 subjects (12,432 men and 15,054 women; follow-up proportion: 66%) underwent the follow-up examinations with a mean follow-up time of 1.6 years. If people participated in the checkups more than once during each survey period (before the disaster, 2008–2010; and after the disaster, 2011-2012), we used the data from the latest year in 2008–2010 and from the earliest year in 2011-2012 in order to examine the direct effect of the disaster on health status. There were some differences in baseline characteristics between individuals who received follow-up examinations and those who did not, such as mean age (66.3 versus 68.0 years) and prevalence of diabetes mellitus (9.3% versus 11.4%) and hypertension (53.9% versus 58.5%), while there were few differences in baseline BMI (23.5 kg/m^2^ versus 23.5 kg/m^2^) and proportions of women (54.8% versus 55.4%) and excessive drinkers (9.3% versus 10.0%).

### 2.2. Ethical Considerations

Informed consent was obtained from the community representatives to conduct an epidemiological study using their data based on guidelines of the Council for International Organizations of Medical Science [2]. The present observational study was approved by the Fukushima Medical University Institutional Review Board (no. 1916). Individuals ≥40 years of age were evaluated using the items in the Specific Health Examination, which is based on Japan's Act on Assurance of Medical Care for Elderly People (Act no. 80, 1982). All participants in the Fukushima Health Management Survey provided written informed consent at our follow-up survey, and these consents have been retained in a secure storage facility.

### 2.3. Patient Characteristics

An interviewer obtained histories for cigarette smoking and weekly alcohol intake, and participants who consumed ≥44 g of ethanol per day were classified as being current excessive drinkers. We examined various patient characteristics—including height; weight; abdominal circumference; body mass index (BMI); blood pressure; and aspartate aminotransferase (AST), alanine aminotransferase (ALT), *γ*-glutamyl transpeptidase (*γ*-GT), triglyceride (TG), high-density lipoprotein cholesterol (HDL-C), low-density lipoprotein cholesterol (LDL-C), HbA1c, fasting plasma glucose, urine protein, and urine sugar levels. Additional measurements included serum creatinine levels, hematocrit levels, hemoglobin levels, estimated glomerular filtration rate, uric acid levels, urine testing for occult blood, and peripheral blood counts, which evaluated the number of red blood cells, platelets, and white blood cells. All HbA1c before and after the disaster were measured using the enzyme method at the laboratory of the Fukushima Preservative Service Association of Health, except for residents in Futaba (*n* = 706). Since the trends in HbA1c before and after the disaster in Futaba were essentially the same as other communities, we included the data of Futaba for analysis. Each subject's HbA1c level was estimated according to the National Glycohemoglobin Standardization Program guidelines, and the equivalent value was calculated using the following equation: HbA1c (%) = 1.019 × HbA1c (%) + 0.30% [3].

According to the Committee of the Japan Diabetes Society on the diagnosis criteria of diabetes [3], we defined the following. (1) Diabetes was defined as a fasting plasma glucose level ≥126 mg/dL (7.0 mmol/L), an HbA1c level ≥6.5%, or the self-reported use of antihyperglycemic agents. (2) Borderline glucose was defined as a fasting plasma glucose level 110−125 mg/dL (6.1–6.9 mmol/L) or an HbA1c level 6.0–6.4%. (3) Normal-high glucose was defined as a fasting plasma glucose level 100–109 mg/dL (5.5–6.0 mmol/L) or an HbA1c level 5.6–5.9%. (4) Normal glucose was defined as a fasting plasma glucose level <100 mg/dL (5.5 mmol/L) and an HbA1c level <5.6%.

### 2.4. Statistical Analysis

Data are presented as mean ± standard deviation. Changes in subject characteristics before and after the disaster were compared using Student's paired *t*-test. Analysis of covariance was used to examine the differences in characteristics of the evacuees and nonevacuees after adjusting for age and sex. The incidence of diabetes among evacuees and nonevacuees after the earthquake was adjusted for age and was compared using the Mann-Whitney *U* test or the *χ* ^2^ test. The hazard ratios (HRs) of incidence of diabetes and 95% confidence intervals (CI) for risk factors were calculated with adjustment for age and other potential confounding factors using the Cox proportional hazards model. SAS version 9.3 (SAS Institute, Cary, North Carolina, USA) was used for analyses. All probability values for statistical tests were 2-tailed, and *P* values < 0.05 were considered statistically significant.

## 3. Results

### 3.1. Clinical Characteristics

The baseline clinical characteristics of the 27,486 subjects (12,432 men and 15,054 women; follow-up rate, 66%; mean follow-up period, 1.5 years) are listed in Table 1. The mean age, BMI, and HbA1c level of the subjects were 66.3 years, 23.5 kg/m^2^, and 5.52%, respectively.

### 3.2. Changes in Clinical Characteristics after the Disaster

**Figure 1:**
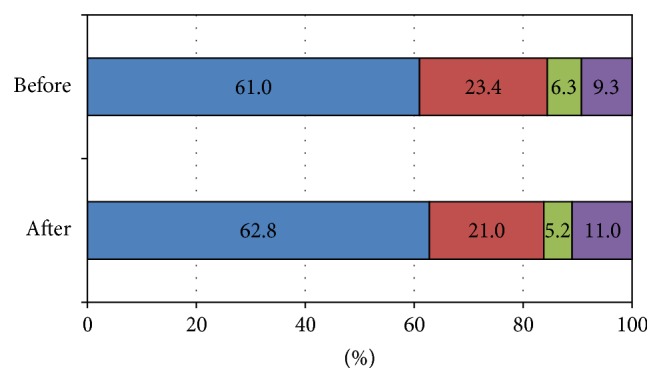
Prevalence of diabetes before and after the Great East Japan Earthquake. Subjects were classified as having normal glucose, normal-high glucose, borderline glucose, or diabetes, in accordance with the Japan Diabetes Society (JDS) guidelines. Purple color: diabetes was defined as a fasting plasma glucose level ≥126 mg/dL (7.0 mmol/L), an HbA1c level ≥6.5%, or the self-reported use of antihyperglycemic agents. Green color: borderline glucose was defined as a fasting plasma glucose level ≥110 mg/dL (6.1 mmol/L), an HbA1c level ≥6.0% with a fasting plasma glucose level <126 mg/dL (7.0 mmol/L), or an HbA1c level <6.5%. Red color: normal-high glucose was defined as a fasting plasma glucose level ≥100 mg/dL (5.5 mmol/L), an HbA1c level ≥5.6% with a fasting plasma glucose level <110 mg/dL (6.1 mmol/L), or an HbA1c level <6.0%. Blue: normal glucose was defined as a fasting plasma glucose level <100 mg/dL (5.5 mmol/L) with an HbA1c level <5.6%.

Subjects were classified as having diabetes, borderline glucose, normal-high glucose, and normal glucose levels at baseline (before the earthquake). The prevalence rates of diabetes and normal glucose, normal-high glucose, and borderline glucose levels before and after the Great East Japan Earthquake are shown in Figure 1. After the disaster, the prevalence of diabetes increased significantly from 9.3% to 11.0% (*P* < 0.0001), and the incidence of normal glucose also increased significantly from 61.0% to 62.8% (*P* < 0.0001; Figure 1).

In the normal glucose group (16,760 subjects; 7,564 men and 9,196 women; mean age, 65.3 years), HbA1c levels increased significantly after the disaster from 5.20% ± 0.21% to 5.21% ± 0.26% (*P* < 0.0001; Table 2). In addition, BMI; blood pressure; and LDL-C, AST, ALT, and *γ*-GT levels increased significantly (all *P* < 0.0001; Table 2), while the HDL-C levels decreased significantly from 60.5 ± 14.8 mg/dL to 59.8 ± 14.8 mg/dL (*P* < 0.0001).

In the normal-high glucose group (6,440 subjects; 2,616 men and 3,824 women; mean age, 67.7 years), HbA1c levels improved significantly after the disaster from 5.67% ± 0.11% to 5.64% ± 0.30% (*P* < 0.0001). In addition, BMI; blood pressure; and AST, ALT, and *γ*-GT levels increased significantly (*P* < 0.0001), while HDL-C levels decreased significantly from 58.4 ± 14.0 mg/dL to 57.6 ± 14.1 mg/dL (*P* < 0.0001). In the borderline glucose group (1,735 subjects; 770 men and 965 women; mean age, 68.2 years), HbA1c levels increased significantly after the disaster from 6.10% ± 0.13% to 6.14% ± 0.61% (*P* = 0.0004; Table 2). In addition, BMI (*P* < 0.0001); diastolic blood pressure (*P* = 0.0002); and AST (*P* = 0.0154), ALT (*P* = 0.0022), and *γ*-GT (*P* < 0.0163) levels increased significantly, while HDL-C levels decreased significantly from 56.8 ± 14.4 mg/dL to 56.1 ± 14.1 mg/dL (*P* < 0.0001).

### 3.3. Change in Clinical Characteristics among Nonevacuees and Evacuees after the Disaster

We also examined the effects of evacuation on glucose metabolism among evacuees and nonevacuees (Table 3). In the normal glucose group, the increase in HbA1c level was significantly greater among evacuees (5,635 subjects; 2,511 men and 3,124 women; mean age, 65.2 years) than among nonevacuees (11,125 subjects; 5,053 men and 6,072 women; mean age, 65.3 years) (*P* < 0.0001). Furthermore, the increases in BMI; diastolic blood pressure; and LDL-C, AST, ALT, and *γ*-GT levels were significantly greater among evacuees than nonevacuees (*P* < 0.0001), while the decrease in HDL-C levels was significantly greater among evacuees than among nonevacuees (*P* < 0.0001).

In the normal-high glucose group, the decrease in HbA1c levels was significantly greater (*P* < 0.0001; Table 3) among evacuees (2,335 subjects; 910 men and 1,425 women; mean age, 67.3 years) than among nonevacuees (4,105 subjects; 1,706 men and 2,399 women; mean age, 67.9 years) (*P* < 0.0001). However, the increase in BMI was significantly greater (*P* < 0.0001) among evacuees than among nonevacuees.

In the borderline glucose group, the increase in HbA1c levels was significantly greater among nonevacuees (1,011 subjects; 454 men and 557 women; mean age, 68.3 years) than among evacuees (724 subjects; 316 men and 408 women; mean age, 68.0 years) (*P* = 0.0007). In addition, the increase in BMI was significantly greater among evacuees than among nonevacuees (*P* < 0.0148).

On the other hand, the proportion of smoking and excessive alcohol drinking did not increase after the disaster in the present study; the proportions of smoking and excessive drinking were 12.7% and 4.5% before the disaster and 11.2% and 3.2% after the disaster, respectively.

### 3.4. Risk Factors for the Incidence of Diabetes after the Disaster

The Cox proportional hazards model was used to identify independent associations between baseline risk factors and the incidence of diabetes after the disaster (Table 4). Evacuation was significantly associated with the incidence of diabetes (hazard ratio (HR), 1.399; *P* < 0.0001) after adjusting for age; sex; BMI; smoking status; systolic blood pressure; and HDL-C, ALT, and *γ*-GT levels. Various factors were significantly associated with the incidence of diabetes, including baseline BMI (HR, 1.177; *P* < 0.00001), systolic blood pressure (HR, 1.008; *P* < 0.001), HDL-C levels (HR, 0.991; *P* = 0.004), ALT levels (HR, 1.006; *P* = 0.001), and *γ*-GT levels (HR, 1.002; *P* = 0.002). Smoking and excessive alcohol drinking status before the disaster were not associated with incidence of diabetes. Further, smoking and excessive drinking status after the disaster were not associated with the incidence of diabetes.

### 3.5. The Incidence of Diabetes after the Disaster

The crude incidence of diabetes in the nondiabetes groups (24,935 subjects) was 3.0% (743 subjects; Table 5). However, the crude incidence of diabetes was 0.5% (85 subjects) in the normal glucose group (16,760 subjects), 3.5% (223 subjects) in the normal-high glucose group (6,440 subjects), and 25.1% (435 subjects) in the borderline glucose group (1,735 subjects). In the nondiabetes group (16,241 nonevacuees and 8,694 evacuees), the incidence of diabetes was significantly greater (*P* = 0.0002; Table 4) among evacuees (3.6%; 313 subjects) than among nonevacuees (2.6%; 430 subjects). Specifically, in the normal glucose group (11,125 nonevacuees and 5,635 evacuees), the incidence of diabetes was significantly greater (*P* = 0.00425) among evacuees (0.7%; 41 subjects) than among nonevacuees (0.4%; 44 subjects). In contrast, there was no significant difference in the incidence of diabetes among the nonevacuees (3.2%; 132 subjects) and evacuees (3.9%; 91 subjects) in the normal-high glucose group (4,105 nonevacuees and 2,335 evacuees). In the borderline glucose group (1,011 nonevacuees and 724 evacuees), there was no significant difference in the incidence of diabetes between nonevacuees (25.1%; 254 subjects) and evacuees (25.0%; 181 subjects).

## 4. Discussion

In the present study, we observed that the prevalence of diabetes significantly increased among evacuees after the Great East Japan Earthquake and the Fukushima Daiichi nuclear disaster. All metabolic factors (BMI, blood pressure, glucose metabolism, lipid metabolism, and liver function) were significantly exacerbated in the normal and borderline glucose groups. In contrast, HbA1c levels in the normal-high glucose group improved significantly after the disaster, while the other metabolic factors (except glucose metabolism) were significantly exacerbated. In addition, the increase in HbA1c levels was significantly greater among evacuees than among nonevacuees in the normal glucose group. Furthermore, the present study revealed that evacuation was significantly associated with the incidence of diabetes and that the incidence of diabetes was significantly higher among evacuees than among nonevacuees. These findings suggest that chronic metabolic health issues such as obesity, type 2 diabetes, hypertension, and dyslipidemia should be carefully monitored and treated after disaster.

Previous studies have reported that natural disasters (e.g., the Great Hanshin-Awaji earthquake) negatively impact glycemic control in diabetic patients [4]. An association between chronic life-threatening stress and reduced metabolic control among diabetic patients has also been suggested [5]. Similar effects have been reported for hypertension, with ambulatory blood pressure indicating that sympathetic activation results in increased blood pressure after life-threatening events, which may subsequently trigger myocardial infarction [6]. The daily life of evacuees is known to increase stress due to changes in privacy, food availability, work assignments, income, and health [5] and an increase in patient stress is known to aggravate diabetes [7]. Although reduced glycemic control during disasters might be caused by a number of factors (e.g., change in diet, reduced physical stress, and psychological stress), no studies have determined whether evacuation itself might have independent effects on glycemic control. Therefore, our data are the first to indicate that the negative effects of the disaster on metabolic factors were greater among the evacuees than among the nonevacuees.

Interestingly, changes in lifestyle are strongly associated with the prevalence of diabetes. The Diabetes Prevention Program (a multicenter, randomized placebo-controlled trial) examined the effects of two interventions on the prevention or delay of onset of type 2 diabetes in high-risk individuals and found that the risk of developing type 2 diabetes was reduced by 58% and 31% in the intensive lifestyle and metformin-treated groups, respectively, compared with the placebo-treated group [8]. Intensive lifestyle intervention was more effective than medication in slowing the progression to diabetes, partially due to the fact that lifestyle modifications provide greater improvements in insulin sensitivity and *β*-cell functions [9]. Changes in physical activity and diet predicted patients' weight loss, which was also associated with a reduced risk of developing type 2 diabetes mellitus. Thus, lifestyle interventions can be an effective tool in preventing or treating insulin resistance and type 2 diabetes [8], given their close relationship with the incidence of type 2 diabetes.

In our normal-high glucose group, HbA1c levels improved significantly after the disaster, while BMI increased significantly. Obesity is a major risk factor for the development of insulin resistance, which can lead to type 2 diabetes, hypertension, and cardiovascular diseases [10]. Thus, subjects in the normal-high glucose group may develop type 2 diabetes in the future, although long-term studies are required to evaluate this outcome.

The United Kingdom Prospective Study recently reported that, among patients with type 2 diabetes, postinterventional microvascular benefits and the emergence of macrovascular risk reduction are associated with earlier improved glycemic control during a 10-year follow-up [11]. The Steno-2 Study also reported a similar outcome during a 5.5-year evaluation of earlier multifactorial risk reduction among patients with type 2 diabetes [12]. In both trials, enhanced risk reduction was observed, despite the loss of intratrial differences in glucose levels. In addition, the Steno-2 Study revealed diminished differences in blood pressure and lipid levels, suggesting the persistent effects of earlier improved metabolic management. Thus, we suggest that periodic health checkups and lifestyle guidance are critical to improving the long-term health of evacuees.

In conclusion, ours is first study to demonstrate that evacuation is associated with the incidence of diabetes. This information could be used during periodic health checkups in the future to make important lifestyle recommendations to evacuees.

## Figures and Tables

**Table 1:** Baseline clinical and biochemical characteristics before the Great East Japan Earthquake.

	Baseline
Number	27,486
Sex (male/female)	12,432/15,054
Age (years)	66.3 ± 9.4
Body weight (kg)	57.1 ± 10.3
BMI (kg/m^2^)	23.5 ± 3.3
Currently smoking (%)	12.7
Excessive alcohol drinking (%)	4.5
Blood pressure (mmHg)	
Systolic	131.6 ± 16.9
Diastolic	77.0 ± 10.3
Fasting plasma glucose (mg/dL)	100.6 ± 21.7
Hemoglobin A1c (%)	5.52 ± 0.64
Triglycerides (mg/dL)	108.2 ± 69.4
HDL-C (mg/dL)	59.4 ± 14.6
LDL-C (mg/dL)	120.6 ± 29.8
AST (IU/L)	25.0 ± 10.5
ALT (IU/L)	20.7 ± 13.3
*γ*-GT (IU/L)	33.2 ± 46.2

Data are presented as mean ± standard deviation.

BMI, body mass index; HDL-C, high-density lipoprotein cholesterol; LDL-C, low-density lipoprotein cholesterol; AST, aspartate aminotransferase; ALT, alanine aminotransferase; *γ*-GT, *γ*-glutamyl transpeptidase.

**Table 2:** Subject characteristics of subjects classified by diabetic status before and after the Great East Japan Earthquake.

	Normal glucose group			Normal-high glucose group			Borderline glucose group		
	Before	After	*P* value	Before	After	*P* value	Before	After	*P* value
Number	16,760			6,440			1,735		
Sex (male/female)	7,564/9,196			2,616/3,824			770/965		
Age (years)	65.3 ± 9.9	66.8 ± 9.9		67.7 ± 8.6	69.2 ± 8.6		68.2 ± 8.4	69.7 ± 8.4	
Body weight (kg)	56.2 ± 9.9	57.0 ± 10.3	<0.0001	57.2 ± 10.3	57.8 ± 10.6	<0.0001	59.3 ± 10.8	59.6 ± 10.9	0.0003
BMI (kg/m^2^)	23.0 ± 3.0	23.4 ± 3.2	<0.0001	23.9 ± 3.3	24.2 ± 3.4	<0.0001	24.7 ± 3.5	24.8 ± 3.6	<0.0001
Blood pressure (mmHg)									
Systolic	130.4 ± 16.8	132.4 ± 16.2	<0.0001	132.5 ± 16.6	134.2 ± 15.7	<0.0001	134.7 ± 17.7	135.3 ± 16.0	0.202
Diastolic	77.0 ± 10.2	78.3 ± 10.1	<0.0001	77.1 ± 10.4	78.2 ± 9.8	<0.0001	77.5 ± 10.5	78.4 ± 10.2	0.0002
Hemoglobin A1c (%)	5.20 ± 0.21	5.21 ± 0.26	<0.0001	5.67 ± 0.11	5.64 ± 0.30	<0.0001	6.10 ± 0.13	6.14 ± 0.61	0.004
Fasting plasma glucose (mg/dL)	89.8 ± 5.7	93.1 ± 8.2	<0.0001	98.3 ± 6.8	100.8 ± 10.9	<0.0001	110.1 ± 8.8	113.1 ± 16.7	<0.0001
Triglycerides (mg/dL)	103.4 ± 63.1	110.5 ± 67.2	<0.0001	111.8 ± 74.2	119.6 ± 77.1	<0.0001	123.5 ± 69.8	130.0 ± 84.1	<0.0001
HDL-C (mg/dL)	60.5 ± 14.8	59.8 ± 14.8	<0.0001	58.4 ± 14.0	57.6 ± 14.1	<0.0001	56.8 ± 14.4	56.1 ± 14.4	0.0001
LDL-C (mg/dL)	119.8 ± 29.3	122.2 ± 30.6	<0.0001	123.3 ± 30.1	125.0 ± 30.8	<0.0001	123.6 ± 31.0	124.2 ± 31.6	0.675
AST (IU/L)	24.8 ± 10.1	25.3 ± 11.0	<0.0001	24.9 ± 9.1	25.5 ± 10.6	<0.0001	26.6 ± 15.1	27.1 ± 13.7	0.0154
ALT (IU/L)	19.7 ± 11.7	21.0 ± 14.1	<0.0001	20.7 ± 13.3	22.1 ± 14.6	<0.0001	24.1 ± 19.7	25.5 ± 19.5	0.0022
*γ*-GT (IU/L)	32.7 ± 47.2	34.5 ± 47.9	<0.0001	30.5 ± 33.5	32.5 ± 41.7	<0.0001	38.2 ± 62.2	37.9 ± 49.2	0.0163

Data are presented as mean ± standard deviation.

BMI, body mass index; HDL-C, high-density lipoprotein cholesterol; LDL-C, low-density lipoprotein cholesterol; AST, aspartate aminotransferase; ALT, alanine aminotransferase; *γ*-GT, *γ*-glutamyl transpeptidase.

**Table 3:** Changes in clinical and biochemical characteristics among nonevacuees and evacuees after the Great East Japan Earthquake.

	Nonevacuees			Evacuees			*P* values^*∗*^
	Before	After	*P* value	Before	After	*P* value	
Normal glucose group							
Number	11,125			5,635			
Sex (male/female)	5,053/6,072			2,511/3,124			
Age (years)	65.3 ± 9.7	66.8 ± 9.7		65.2 ± 10.3	66.9 ± 10.3		
Body weight (kg)	56.0 ± 9.8	56.4 ± 10.1	<0.0001	56.6 ± 10.0	58.0 ± 10.6	<0.0001	<0.0001
BMI (kg/m^2^)	22.9 ± 3.0	23.1 ± 3.1	<0.0001	23.2 ± 3.1	23.8 ± 3.2	<0.0001	<0.0001
Blood pressure (mmHg)							
Systolic	130.2 ± 16.6	132.4 ± 16.3	<0.0001	130.7 ± 17.1	132.3 ± 15.9	<0.0001	0.6702
Diastolic	77.2 ± 10.1	78.2 ± 10.2	<0.0001	76.6 ± 10.3	78.6 ± 10.0	<0.0001	0.0099
Hemoglobin A1c (%)	5.20 ± 0.20	5.21 ± 0.25	<0.0001	5.19 ± 0.21	5.22 ± 0.26	<0.0001	<0.0001
HDL-C (mg/dL)	60.3 ± 14.8	60.2 ± 14.8	0.1471	60.9 ± 14.8	58.9 ± 14.8	<0.0001	<0.0001
LDL-C (mg/dL)	120.3 ± 29.2	121.3 ± 29.9	<0.0001	118.9 ± 29.6	124.2 ± 31.8	<0.0001	0.0005
AST (IU/L)	24.6 ± 9.0	25.1 ± 11.1	<0.0001	25.1 ± 12.0	25.8 ± 10.9	0.0003	0.0021
ALT (IU/L)	19.4 ± 10.6	20.3 ± 12.8	<0.0001	20.2 ± 13.7	22.5 ± 16.1	<0.0001	<0.0001
*γ*-GT (IU/L)	32.3 ± 45.7	33.5 ± 48.8	<0.0001	33.5 ± 49.9	36.3 ± 45.8	<0.0001	0.0002

Normal-high glucose group							
Number	4,105			2,335			
Sex (male/female)	1,706/2,399			910/1,425			
Age (years)	67.9 ± 8.3	69.3 ± 8.3		67.3 ± 9.1	68.8 ± 9.1		
Body weight (kg)	57.0 ± 10.1	57.3 ± 10.4	<0.0001	57.5 ± 10.5	58.7 ± 10.9	<0.0001	<0.0001
BMI (kg/m^2^)	23.8 ± 3.3	24.0 ± 3.4	<0.0001	24.0 ± 3.3	24.6 ± 3.5	<0.0001	<0.0001
Blood pressure (mmHg)							
Systolic	132.3 ± 16.0	134.4 ± 15.8	<0.0001	132.9 ± 17.7	133.8 ± 15.5	0.018	0.3446
Diastolic	77.5 ± 10.4	78.2 ± 9.8	<0.0001	76.6 ± 10.3	78.2 ± 9.7	<0.0001	0.0861
Hemoglobin A1c (%)	5.66 ± 0.11	5.64 ± 0.27	<0.0001	5.68 ± 0.11	5.63 ± 0.34	<0.0001	<0.0001
HDL-C (mg/dL)	57.9 ± 13.8	57.8 ± 14.1	0.5727	59.4 ± 14.2	57.3 ± 14.0	<0.0001	0.2394
LDL-C (mg/dL)	123.6 ± 30.2	123.9 ± 30.5	0.4586	122.7 ± 30.1	126.9 ± 31.3	<0.0001	0.322
AST (IU/L)	24.9 ± 9.1	25.3 ± 10.1	0.0097	24.8 ± 9.0	26.0 ± 11.4	0.0003	0.4519
ALT (IU/L)	20.6 ± 13.9	21.4 ± 13.6	0.0002	20.9 ± 12.2	23.3 ± 16.1	<0.0001	0.1371
*γ*-GT (IU/L)	30.9 ± 35.1	32.2 ± 44.5	0.0071	29.9 ± 30.6	33.1 ± 36.1	<0.0001	0.0012

Borderline glucose group							
Number	1,011			724			
Sex (male/female)	454/557			316/408			
Age (years)	68.3 ± 8.1	69.8 ± 8.1		68.0 ± 8.8	69.5 ± 8.8		
Body weight (kg)	59.3 ± 11.0	59.3 ± 11.1	0.791	59.4 ± 10.3	60.0 ± 10.6	<0.0001	0.007
BMI (kg/m^2^)	24.7 ± 3.6	24.7 ± 3.7	0.0986	24.6 ± 3.3	25.0 ± 3.4	<0.0001	0.0148
Blood pressure (mmHg)							
Systolic	134.0 ± 17.1	135.7 ± 16.2	0.001	135.8 ± 18.6	134.6 ± 15.7	0.1104	0.54
Diastolic	77.7 ± 10.5	78.2 ± 10.2	0.1609	77.1 ± 10.4	78.8 ± 10.1	<0.0001	0.4414
Hemoglobin A1c (%)	6.10 ± 0.13	6.16 ± 0.55	0.0002	6.11 ± 0.13	6.12 ± 0.68	0.6701	0.0007
HDL-C (mg/dL)	56.4 ± 14.5	56.2 ± 14.5	0.3228	57.5 ± 14.3	55.9 ± 14.2	<0.0001	0.7792
LDL-C (mg/dL)	122.7 ± 30.5	122.4 ± 30.5	0.7321	125.3 ± 32.0	126.7 ± 33.0	0.3006	0.9227
AST (IU/L)	26.5 ± 12.7	26.4 ± 12.2	0.9944	26.7 ± 17.9	28.1 ± 15.5	0.0004	0.7899
ALT (IU/L)	24.1 ± 19.6	24.0 ± 17.5	0.7902	24.2 ± 19.8	27.5 ± 21.9	<0.0001	0.8524
*γ*-GT (IU/L)	37.2 ± 53.7	37.0 ± 55.6	0.3544	39.7 ± 72.5	39.2 ± 38.3	<0.0001	0.0973

Data are presented as mean ± standard deviation.

^*∗*^Age-adjusted *P* value comparing the changes in the evacuee group to the changes in the nonevacuee group before and after the earthquake.

BMI, body mass index; HDL-C, high-density lipoprotein cholesterol; LDL-C, low-density lipoprotein cholesterol; AST, aspartate aminotransferase; ALT, alanine aminotransferase; *γ*-GT, *γ*-glutamyl transpeptidase.

**Table 4:** Hazard ratios (HR) and 95% confidence intervals (CI) of diabetes for risk factors after the Great East Japan Earthquake: the Fukushima Health Management Survey 2008–2012.

Model	HR (95% CI)	*P* value
Evacuation	1.399 (1.203–1.628)	<0.0001
Age (years)	1.023 (1.014–1.032)	<0.0001
Sex (0, women; 1, men)	1.566 (1.328–1.847)	<0.0001
BMI (kg/m^2^)	1.177 (1.151–1.203)	<0.0001
Excessive drinking (0, no; 1, yes)	0.973 (0.692–1.368)	0.88
Currently smoking (0, no; 1, yes)	1.178 (0.944–1.470)	0.15
Systolic blood pressure (mmHg)	1.008 (1.004–1.013)	<0.001
LDL-C (mg/dL)	1.000 (0.998–1.003)	0.79
HDL-C (mg/dL)	0.991 (0.986–0.997)	0.004
ALT (IU/L)	1.006 (1.002–1.010)	0.001
*γ*-GT (IU/L)	1.002 (1.001–1.003)	0.002

Data are presented as hazards ratio (95% confidence interval).

HR, hazard ratio; CI, confidence interval; BMI, body mass index; LDL-C, low-density lipoprotein cholesterol; HDL-C, high-density lipoprotein cholesterol; AST, aspartate aminotransferase; ALT, alanine aminotransferase; *γ*-GT, *γ*-glutamyl transpeptidase.

**Table 5:** The incidence of diabetes after the Great East Japan Earthquake.

Before the earthquake	Incidence of diabetes after the earthquake (*N*)			*P* value^*∗*^
	Total	Nonevacuees	Evacuees	
Nondiabetic type (*N* = 24,935)	3.0% (743)	2.6% (430)	3.6% (313)	0.00002

Normal type (*N* = 16,760)	0.5% (85)	0.4% (44)	0.7% (41)	0.004
Normal-high type (*N* = 6,440)	3.5% (223)	3.2% (132)	3.9% (91)	0.15
Borderline type (*N* = 1,735)	25.1% (435)	25.1% (254)	25.0% (181)	0.95

Data are presented as percentage (number).

^*∗*^ *P* value comparing the incidence of diabetes after the earthquake between the evacuee group and the nonevacuee group.

## Abbreviations

HbA1c: Hemoglobin A1c
BMI: Body mass index
AST: Aspartate aminotransferase
ALT: Alanine aminotransferase
*γ*-GT: *γ*-Glutamyl transpeptidase
TG: Triglyceride
HDL-C: High-density lipoprotein cholesterol
LDL-C: Low-density lipoprotein cholesterol
Cr: Serum creatinine
eGFR: Estimated glomerular filtration rate
UA: Uric acid
RBC: Red blood cell count
Ht: Hematocrit
Hb: Hemoglobin
WBC: Platelet count and white blood cell count.
