# Corrigendum to “Evacuation after the Fukushima Daiichi Nuclear Power Plant Accident Is a Cause of Diabetes: Results from the Fukushima Health Management Survey”

**DOI:** 10.1155/2015/415253

**Published:** 2015-08-13

**Authors:** Hiroaki Satoh, Tetsuya Ohira, Mitsuaki Hosoya, Akira Sakai, Tsuyoshi Watanabe, Akira Ohtsuru, Yukihiko Kawasaki, Hitoshi Suzuki, Atsushi Takahashi, Gen Kobashi, Kotaro Ozasa, Seiji Yasumura, Shunichi Yamashita, Kenji Kamiya, Masafumi Abe

**Affiliations:** ^1^Department of Nephrology, Hypertension, Diabetology, Endocrinology, and Metabolism, Fukushima Medical University, Fukushima 960-1295, Japan; ^2^Radiation Medical Science Center for the Fukushima Health Management Survey, Fukushima Medical University, Fukushima 960-1295, Japan; ^3^Department of Epidemiology, Fukushima Medical University, Fukushima 960-1295, Japan; ^4^Department of Pediatrics, Fukushima Medical University, Fukushima 960-1295, Japan; ^5^Department of Radiation Life Sciences, Fukushima Medical University, Fukushima 960-1295, Japan; ^6^Department of Radiation Health Management, Fukushima Medical University, Fukushima 960-1295, Japan; ^7^Department of Cardiology and Hematology, Fukushima Medical University, Fukushima 960-1295, Japan; ^8^Department of Gastroenterology and Rheumatology, Fukushima Medical University, Fukushima 960-1295, Japan; ^9^Department of Planning and Management, National Institute of Radiological Sciences, Fukushima Medical University, Fukushima 960-1295, Japan; ^10^Department of Epidemiology, Radiation Effects Research Foundation, Fukushima Medical University, Fukushima 960-1295, Japan; ^11^Department of Public Health, Fukushima Medical University, Fukushima 960-1295, Japan; ^12^Atomic Bomb Disease Institute, Nagasaki University, Nagasaki, Japan; ^13^Research Institute for Radiation Biology and Medicine, Hiroshima University, Hiroshima, Japan

In the paper titled “Evacuation after the Fukushima Daiichi Nuclear Power Plant Accident Is a Cause of Diabetes: Results from the Fukushima Health Management Survey” [1], the description of “multivariate logistic regression analysis” (Line 11) in the Abstract is incorrect. “Cox proportional hazards model” is the correct form mentioned in the description in Materials and Methods.
